# Preparation of a Chemically Reduced Graphene Oxide Reinforced Epoxy Resin Polymer as a Composite for Electromagnetic Interference Shielding and Microwave-Absorbing Applications

**DOI:** 10.3390/polym10111180

**Published:** 2018-10-23

**Authors:** Ahmad Fahad. Ahmad, Sidek Ab Aziz, Zulkifly Abbas, Suzan Jabbar Obaiys, Ahmad Mamoun Khamis, Intesar Razaq Hussain, Mohd Hafiz Mohd Zaid

**Affiliations:** 1 Department of Physics, Faculty of Science, Universiti Putra Malaysia, Serdang 43400, Malaysia; za@upm.edu.my (Z.A.); akhameis@yahoo.com (A.M.K.); mhmzaid@upm.edu.my (M.H.M.Z.); 2 School of Mathematical & Computer Sciences, Heriot-Watt University Malaysia, Putrajaya 62200, Malaysia; s.obaiys@hw.ac.uk; 3 Department of Chemical and Environmental Engineering, Universiti Putra Malaysia, Serdang 43400, Malaysia; Intesar_Hu85@yahoo.com

**Keywords:** reduced graphene oxide, epoxy resins, permittivity, permeability, Cole–Cole

## Abstract

The preparation of chemically reduced graphene oxide (rGO) and the optimization of epoxy resins’ properties using micro or nanofillers are now common practices. rGO nanoparticles (60 nm) based on an epoxy resin polymer were prepared at the concentrations of 0, 1, 2, 3, 4, and 5% weight percentage with fixed 6-mm thicknesses. The dielectric properties of the composites were measured by the reflection/transmission technique in connection with a vector network analyser (VNA) at a frequency range of 8–12 GHz. The microwave absorption and shielding effectiveness properties were calculated by using the reflection S_11_ and transmission S_21_ results. The microstructure and morphology of the polymer and the rGO/cured epoxy composites were studied by field emission scanning electron microscopy (FE-SEM), Fourier-transform infrared (FT-IR) spectroscopy, and the X-ray Diffraction (X-RD) technique for characterizing crystalline materials. The dielectric and other properties of the rGO/cured epoxy composites were investigated based on the filler load and frequency. It was found that the applied frequency and the filler concentrations affected the dielectric properties of the rGO/cured epoxy composites. The results showed that the introduction of rGO particles to the composites increased their dielectric properties smoothly. The study of the dependence on frequency of both the dielectric constant ε′ and the dielectric loss ε″ showed a decrease in both quantities with increasing frequency, indicating a normal behaviour of the dielectrics. Cole–Cole plots were drawn with ε′ and ε″. A theoretical simulation in terms of the Cole–Cole dispersion law indicates that the Debye relaxation processes in the rGO/cured epoxy composites are improved due to the presence of the rGO filler. Moreover, with the addition of rGO as a filler into the Epoxy matrix, it now exhibits promise as a lightweight material for microwave absorption as well as an effective electromagnetic interference (EMI) shielding material.

## 1. Introduction

Electromagnetic interference (EMI) is one of the most undesirable by-products of telecommunication devices and high-frequency electronic systems. Any device that utilizes, processes, transmits, or distributes electrical energy of any form may emit signals of an electromagnetic nature and interfere with nearby systems and equipment in their normal operation [1]. Such signals may also have negative effects on human health. Efforts to reduce pollution of an electromagnetic nature have been made with the use of EMI shielding materials [2]. Signals are attenuated by such materials through absorption and/or reflection of the radiation power [3]. Traditionally, magnetic materials and metals have been utilized for the shielding of EMI due to their good mechanical properties and high effectiveness in shielding. However, among the drawbacks that these materials have, they are heavy, susceptible to corrosion, and difficult to process. The metals’ high conductivity also limits their application as absorbers of electromagnetic waves due to their shallow skin depth; thus, almost all of the power is reflected on the surface [4]. There is an increasing need to develop practical and effective EMI shielding materials that overcome the conventional metal-based shields’ shortcomings, which has increased the interest of researchers in these novel materials [5].

Polymer composites for applications in EMI shielding have attracted the attention of many researchers. This is mainly because of their characteristics, which usually include low density, good mechanical properties, high dimensional and thermal stability, and also special and less common features, such as electromagnetic absorption [6]. Also, the disadvantages that are associated with the use of metals, including corrosion resistance, light weight, flexibility, as well as processing difficulty, are addressed by these materials [7]. carbon black [8], Carbon-based particles, such as carbon fibre [9], and carbon nanotubes [10], have been investigated as effective fillers for preparing absorbing materials and conducting polymer composites for EMI shielding applications, military affairs, commerce, and electronic instruments in industry. Most of the materials that are used for microwave absorption are composed of powders of magnetic loss, which include nickel, ferrite, and cobalt, and materials for dielectric loss, such as conducting polymers and carbon nanotubes. Although the carbon nanotubes’ ability for microwave absorption is extremely weak, previous reports have indicated that magnetic material and carbon nanotube composites would exhibit excellent properties for microwave absorption [11].

Recently, graphene’s microwave absorption and electromagnetic shielding abilities have been investigated. Various materials with graphene particles have therefore been highlighted [12] and have shown interesting electromagnetic absorption outcomes. For instance, the improved electromagnetic wave absorption results of chemically reduced graphene oxide (rGO) with residual defects via additional relaxation processes, namely dielectric, polarization, and dipole relaxations, showed that a 6.9 dB microwave reflection loss had been achieved at 7 GHz [12]. Graphene-poly methyl methacrylate nanocomposite microcellular foams were found to have high EMI shielding effectiveness (13–19 dB at 8–12 GHz) by the support of multi-reflections and the scattering of the incident microwaves into the foam samples [13]. Graphene layers with polyaniline nanorods embedded in a paraffin matrix exhibited a microwave reflection loss that was lower than 20 dB from 7.0 to 17.6 GHz when the Debye relaxation process was amended [14]. Graphene nanoplatelets in epoxy resin exhibited a 14.5 dB maximum reflection loss at 18.9 GHz, which was mainly attributed to the charge multipoles at the polarized interfaces into the composite material [15]. Also, graphene was incorporated into an epoxy matrix to study EMI shielding phenomena at the (X-band) frequency, and a 21 dB shielding efficiency was achieved for a 15 wt % (8.8 vol %) loading, indicating that it may be used as an effective and light weight EMI shielding material [16].

Furthermore, reduced graphene oxide has been incorporated into epoxy resin. The results showed shielding effectiveness (SE) values that were higher than the target value (20 dB). As it turned out, at a lower filler loading, and if suitably modified, the surface of the graphene preserves its conductivity, and an EMI SE for practical applications can be achieved [17]. On the other hand, there is a lack of proper research about rGO’s electromagnetic wave absorbing property. rGO shows an improvement in microwave absorption when compared to carbon nanotubes and graphite, is expected to present superior absorption to high-quality graphene, and exhibits promise as microwave absorbing material [18]. Nevertheless, there are some studies that have investigated, and contributed to the knowledge of, the effect of other factors, such as the particle size of the graphene oxide, on the electromagnetic properties, where the properties can be tuned by controlling the size of the graphene particles. The more the particle size increases, the more the dielectric constant increases. The reason for this might be associated with the discrepancy in the interfacial polarization effect that results from different particle sizes [19].

Organic polymer materials, such as epoxy resins, have excellent adhesion to different materials, high resistance to chemical attacks, and excellent mechanical and electrical properties. Besides this, the hardener HY951 provides the best binding property with an epoxy resin [20]. In addition to the abovementioned properties of epoxy resins, they are used in this research due to their diverse characteristics, such as high strength, good stiffness, good thermal stability, antibacterial properties, low contractibility, and strong adherence chemical resistance. They are used widely in industrial applications, such as coatings, adhesives, aerospace structures, electronics, potting, composites, laminates, and the encapsulation of semiconductor devices. Because of their excellent and attractive mechanical and chemical properties, epoxies are the dominant matrix material for structural composites of light-weight polymer–matrix [21]. There have been so many reports on the blending of epoxies with fillers in recent years [22], including the incorporation of nanoferrites, nanoferroelectrics, carbon nanoparticles, single-walled carbon nanotubes (SWNTs), and multi-walled carbon nanotube (MWNTs) into epoxy resins as an absorbing material for EM waves [23].

The present study has two main goals: (a) to prepare chemically reduced graphene oxide (rGO) due to its scalability and high yield, and (b) to prepare rGO/cured epoxy composites and then investigate their structure, morphology, and dielectric properties. The study of the composites’ physical and chemical properties were performed through Fourier-transform infrared spectroscopy (FT-IR) and X-ray diffraction (XRD). Field-emission scanning electron microscopy (FESEM) was conducted to study the structure of the composite.

## 2. Experimental Details

### 2.1. Materials

The materials used in this work are: a polymer matrix (epoxy resin LY1316) and hardener (HY1208, supplied by Buehler, Lake Bluff, IL, USA), Graphite Oxide (prepared by using a modified Staudenmaier method), and reduced Graphite Oxide (manufactured by utilizing a weak base, namely Ammonia (NH_3_) as the reducing agent). The NH_3_ was supplied by Sigma Aldrich (Sarasota, FL, USA).

### 2.2. Preparation of Reduced Graphene Oxide (rGO)

**Figure 1:**
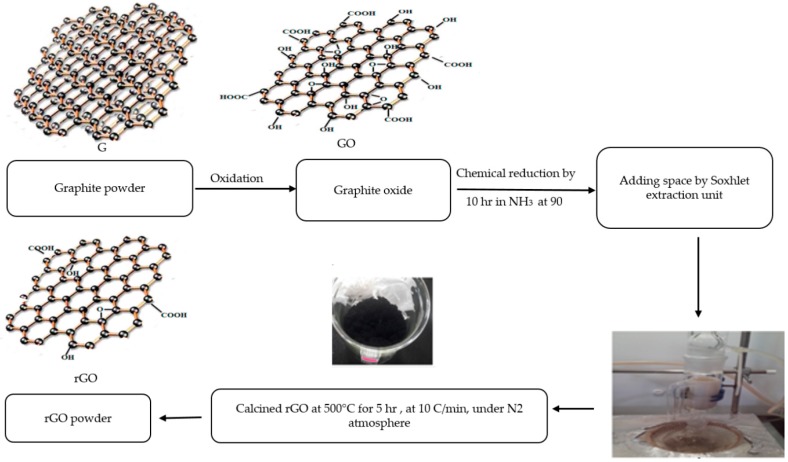
Schematic drawings for the preparation of reduced graphene oxide paper and powder by using the spaced method in a soxhlet unit. GO, Graphite Oxide; rGO, reduced GO.

The preparation method for the synthesis and chemical reduction of rGO paper and then powder involved two major steps. The first step is the synthesis of Graphite Oxide (GO) using the Staudenmaier Method. To perform the reduction process of GO to rGO, about 400 mg of the obtained GO was placed in a cellulose extraction thimble (30 by 100 mm) and then placed in the Soxhlet extraction unit. Approximately 150 mL of 30% Ammonia solution (NH_3_) was used as a reducing agent. The heating temperature was set at 90 °C, and the exposure period was investigated (namely 5 h) when the GO powder had direct contact with the ammonia vapour as well as the condensed liquid. The experimental setup and measurement is as shown in Figure 1.

### 2.3. Preparation of rGO/Cured Epoxy Composites

**Figure 2:**
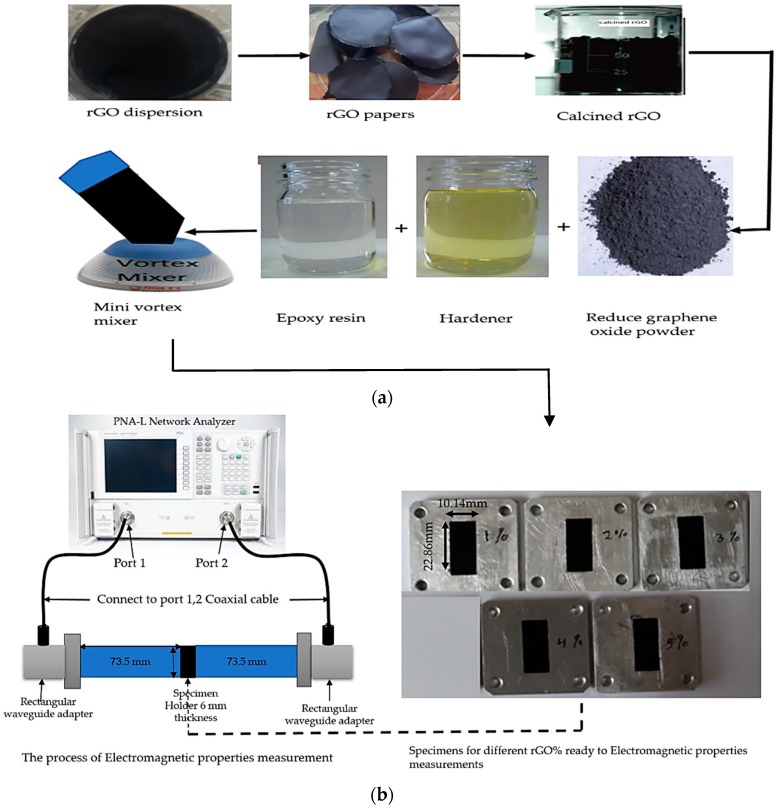
The process for (**a**) The preparation of the rGO/cured epoxy composites and (**b**) The measurement of the electromagnetic properties.

The composites were manufactured in this work using three kinds of materials: epoxy resin (Er), hardener (H), and reduced graphene oxide (rGO), for different filler and polymer percentages which is clearly presented in Table 1. Er, H, and rGO were mixed using a mini-mechanical vortex mixer for 15 min to homogenize the resulting materials. The mixture was poured into rectangular aluminum molds with a thickness of 6 mm, and the coating was allowed to cure in air for 48 h or by utilizing an oven at a temperature between 80 and 140 °C. Figure 2a,b shows the specimens’ preparation process and the measurement of the electromagnetic properties.

### 2.4. Characterisation

All of the characterizations of the pure material under study (rGO, epoxy resin, hardener, and the cured epoxy composites) at different percentages of filler were carried out as follows.

#### 2.4.1. X-ray Diffraction (XRD)

The analysis of the samples’ phase structure was performed using X-ray diffraction (XRD, XD-3, Cu Ka radiation) under ambient conditions with a Lynx Eye detector using a Bruker diffractometer (Yuseong, Daejeon, Korea) over a 5–90° 2θ range. Cu-Kα radiation with a wavelength of 1.54 Å (nickel filtered) was the X-ray beam and operated at 40 kV generator voltage and 35 mA current value settings. The rGO samples were in the form of a fine powder, while the rGO/cured epoxy composite samples were cut from the specimens that were prepared in advanced in a solid form.

#### 2.4.2. Fourier-Transform Infrared (FT-IR) Spectroscopy

In order to obtain appropriate information on the functional groups that were present in the modified reduced graphene oxide, epoxy/hardener, and rGO/cured epoxy composites, an FT-IR analysis was conducted. The spectra were measured by mixing about 0.05–0.1 wt % of the fine ground modified graphite oxide samples with KBr powder, which was then compressed to form a pellet. Using the micro die method, the FTIR spectra of the pellet were measured using a Perkin-Elmer spectrum (Waltham, MA, USA) 100 at 400–4000 cm^−1^.

#### 2.4.3. Field Emission Scanning Electron Microscopy (FE-SEM)

The morphology of the prepared rGO/cured epoxy composite samples was studied and observed by field-emission scanning electron microscopy (FE-SEM, JEOL JSM-7001F) with a field emission cathode backscatter detector and a resolution of 1 nm at 30 kV up to a 700, 0009× magnification and using an Accelerator voltage between 0.1 and 30 kV. All composite samples were coated with gold before analysis.

#### 2.4.4. Electrical Properties

The s-scattering parameters of the reflection (S_11_) and transmission (S_21_) coefficients were measured, and the dielectric properties of the rGO/cured epoxy composites at room temperature in the frequency range of 8–12 GHz were investigated by utilizing transmission line techniques. A rectangular specimen were inserted into a 22.86 × 10.14 × 6 mm^3^ aluminum sample holder that was connected between the waveguide flanges of an Agilent E8362B network analyser. A full two-port calibration was performed along with the sample holder to default any loss and power redistribution due to the sample holder. All of the composite samples were tested, and, for each sample, 201 data points were taken within the specified frequency range.

#### 2.4.5. EMI Shielding Property

##### EMI Shielding Mechanisms

The electromagnetic interference (EMI) shielding was calculated by utilizing the S-parameter results [24]. Electromagnetic interference (EMI) shielding is defined as the attenuation of electromagnetic radiation by reflection and/or absorption of the incident power. The incident electromagnetic radiation on a shield can be resolved into three parts; namely, transmittance (T), absorption (A), and reflection (R), with the sum (T + A + R) equal to 1. Hence, the SE _total_ is the sum of contributions from the absorption loss (SE_A_), the reflection loss (SE_R_), and multiple reflections (SEM), i.e.,

SE _total_ = SE_A_ + SE_R_ + SE_M_. (1)

The SE _total_ of a shielding material can be written as

SE _total_ = 10 log_10_ (1/S_21_^2) (2)

where S_21_^2 is the transmittance value (T), which can be measured from −(P_i_/P_t_) [25]. The P_i_ and P_t_ are, respectively, the incident and transmitted powers, considering the effective absorbance (A _eff_), which is defined as

A _eff_ = (1 − S_11_^2 − S_21_^2)/(1 − S_11_^2). (3)

With regard to the power of the incident electromagnetic wave inside the shielding material, the SE _total_ can be rewritten and described as the sum of the two terms of effective absorbance and reflectance:

SE _total_ = 10 log_10_ (1/(1 − S_11_^2)) + 10 log_10_ ((1 − S_11_^2)/S_21_^2 = SE_R_ + SE_A_. (4)

Using these equations, the total SE was resolved into absorption and reflection loss.

## 3. Results and Discussion

### 3.1. Powder X-ray Diffraction

**Figure 3:**
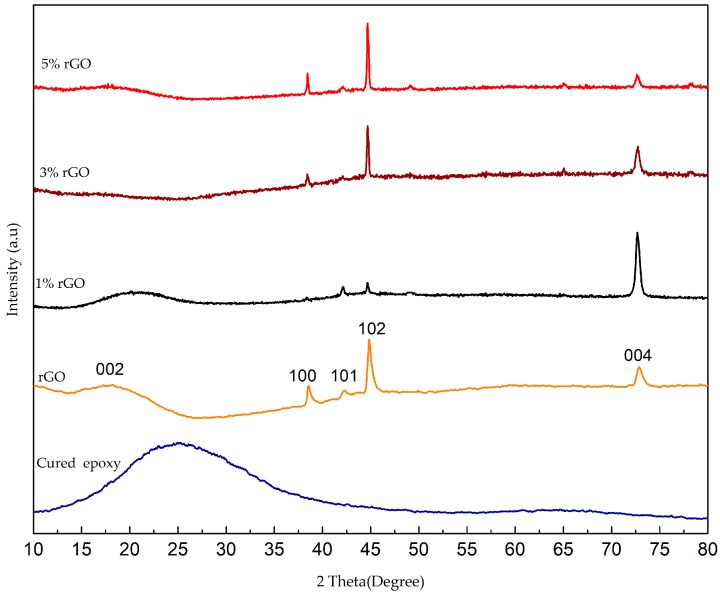
The XRD patterns of cured epoxy, rGO, and rGO/cured epoxy at different percentages of rGO.

X-ray diffraction measurements were utilized to investigate the phase composition and the crystalline structure of the synthesized samples. Figure 3 illustrates the X-ray diffraction pattern of the cured epoxy, the rGO, and the rGO/cured epoxy composites at the filler weight percentages of 1%, 3%, and 5%. The XRD spectra of the neat epoxy showed the appearance of a wide diffraction from 10° to 45° of a broad amorphous peak at an angle of 25.04°. The observed diffraction peak is caused by the scattering of the cured epoxy molecules, which reveals its amorphous nature [26]. Also, Figure 3 clarifies the X-ray diffraction pattern of the rGO powder, showing good crystallinity. The curve indicates a series of diffraction peaks at 2θ = 17.85°, 38.57°, 42.23°, 44.87°, and 73.02°, which corresponds to the (002), (100), (101), (102), and (004) planes, respectively, of the hexagonal carbon structure and crystal planes (JCPDS no. 19-0629) [27]. A crystalline behaviour of rGO having the highest peak appeared at an angle of 44.87°, which indicates a high degree of crystallinity. The degree of crystallinity is the most important basic parameter for characterizing crystalline polymers. On the other hand, Figure 3 shows the diffraction pattern of the rGO/cured epoxy composite containing 1%, 3%, and 5% filler by weight of resin. X-ray diffraction revealed that the composite was crystalline, as the highest peaks were observed at the angles of 72.63°, 44.71°, and 44.67°. Another interesting observation from Figure 3 was the intensity beside the obvious broad diffraction peak at 2θ = 18.18. Because of the amorphous state of the cured epoxy structure in the composite, the intensity of this diffraction peak is relatively weak. The change of the characteristic peak for rGO particles in the rGO/cured epoxy composites can be correlated to fully exfoliate the rGO particles in the polymer matrix. The composite shows a varying diffraction pattern for each rGO percentage, which could be due to the homogeneous dispersion and complete exfoliation of rGO in the cured epoxy matrix. It is clear that the diffraction peak of the 5% rGO/cured epoxy composite at 2θ = 73.02° became less sharp when compared with the 1% and 3% rGO/cured epoxy composites.

The average crystallite size of the rGO nanoparticles (≈60 nm) was calculated by the XRD line broadening using the Scherer equation:

(5) $$D=\frac{k\lambda }{\beta \cos\theta }$$

where *D* is the average crystallite size in nm, k is the shape factor (normally 0.9 for cubic shapes), λ is the wavelength of the X-ray, θ is Bragg’s diffraction angle, and β is the broadening of the diffraction line measured at full-width half-maximum intensity in radians (FWHM data converted to radians).

### 3.2. Field Emission-Scanning Electron Microscopy

**Figure 4:**
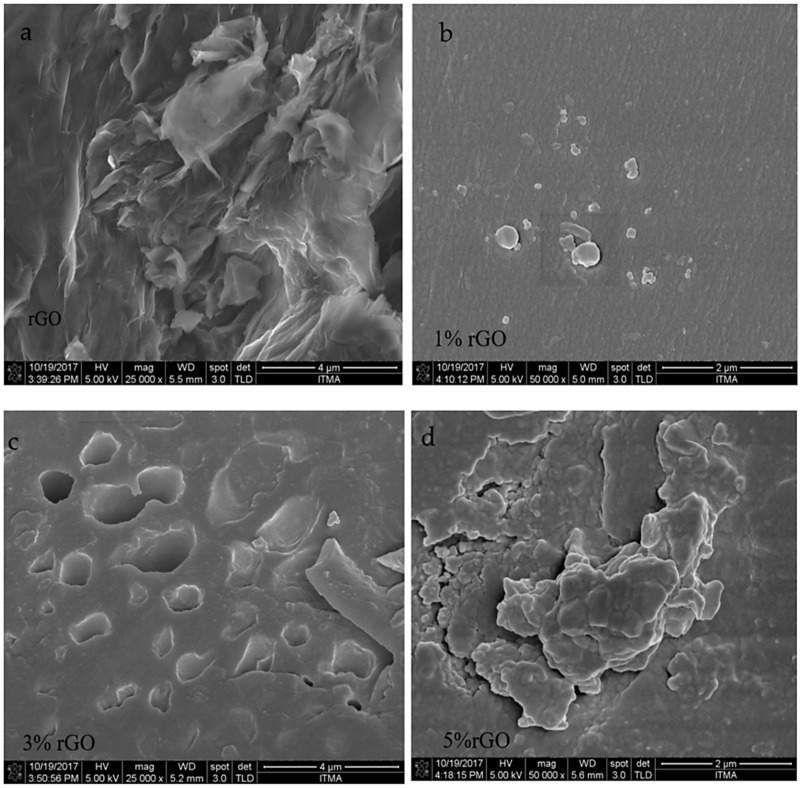
The FE-SEM micrographs of the (**a**) pure rGO and cured epoxy composites with (**b**) 1%, (**c**) 3%, and (**d**) 5% rGO loadings.

The results of Field Emission Scanning electron microscopy (FE-SEM) (structural and morphological characterization) for the rGO as well as the rGO/cured epoxy composites at different rGO loadings (1%, 3%, and 5% rGO) are shown in Figure 4a–d with 25,000×–50,000× magnification. It can be seen from Figure 4a that the pure rGO has a wrinkled, irregular, folding, and many-folded layered structure with a lateral size of several micrometres [28]. The layered structure was formed by rGO particles and it is interesting to see that the layers are continually cross-linked in a flaky textured form without any amorphous or other kinds of crystallized phase particles, as described by [29]. To understand the dispersion of rGO in the epoxy matrix, the FE-SEM image of a rGO/cured epoxy composite is shown in Figure 4b–d. Figure 4b shows the homogeneity of the rGO particles’ dispersion in the cured epoxy resin for the 1% rGO to 99% cured epoxy composite. The rGO particles are observed as small particles that are scattered throughout the polymer matrix of the composite, as well as fully incorporated within the epoxy resin matrix embedded in the polymer matrix. On the other hand, after increasing the percentage of rGO (3% and 5%) in the epoxy composite, the dispersion of rGO powder in the matrix in Figure 4c,d showed obvious differences. Figure 4c shows that the rGO powder was dispersed in the epoxy resin as merged particles of a large size. Figure 4d shows that the rGO powder was dispersed in the epoxy resin in the form of agglomerates. These phenomena are quite different from the rGO dispersion at a low percentage of rGO shown in Figure 4b, suggesting the re-agglomeration of rGO during its addition to the epoxy resin. A similar phenomenon was also found in carbon nanotube (CNT)/epoxy composites [30]. The morphology figures indicate that rGO particles have indeed reacted with the cured epoxy to produce rGO/cured epoxy composites.

### 3.3. The Morphology of the rGO and rGO/Cured Epoxy Composites

**Figure 5:**
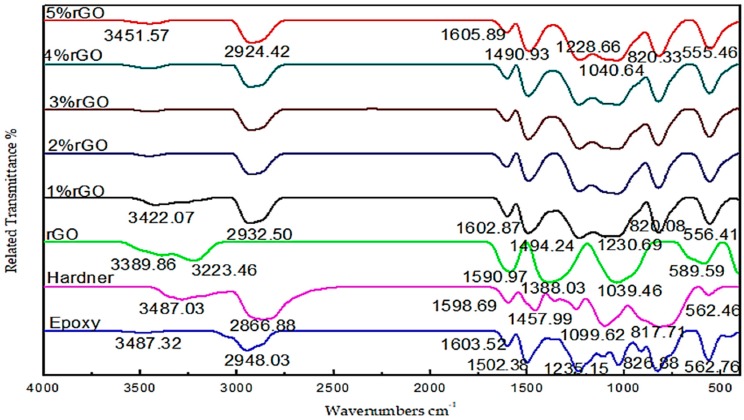
The FT-IR spectra of the neat Epoxy, the Hardener, and the rGO/cured epoxy composites at different percentages of filler.

Fourier-transform infrared (FT-IR) spectroscopy was used to determine the nature of the functional groups present in the surface of the prepared rGO powder, the Epoxy, the Hardener, and the rGO/cured epoxy composites at different percentages of rGO filler. FT-IR spectra were recorded on a Bruker Vertex 70 spectrometer (Waltham, MA, USA) at room temperature (27 ℃) over a frequency range of 400–4000 cm^−1^. Figure 5 presents the typical FT-IR spectra of the rGO powder, the Epoxy, the Hardener, and the rGO/cured epoxy composites. Figure 5 shows absorption bands corresponding to C–O stretching at 1039.46 cm^−1^, C–OH stretching at 1388.03 cm^−1^, phenolic O–H deformation vibrations at 1494.24 cm^−1^, CC stretching at 1590.97 cm^−1^, C=O carbonyl stretching at 3223.46 cm^−1^, and O–H stretching vibrations at 3389.86 cm^−1^ [31,32]. These features strongly prove the presence of carbonyl and carboxyl functional groups on the surface of the rGO flakes [33]. Furthermore, Figure 5 shows absorption bands corresponding to O–H stretching vibrations at 3487.32 cm^−1^, C–H of CH_2_ stretching at 2948.03 cm^−1^, C=C stretching at 1603.52 cm^−1^, aromatic C–C stretching at 1502.38 cm^−1^, C–O–C stretching at 1235.15 cm^−1^, C–O stretching at 826.88 cm^−1^, and rocking CH_2_ at 562.76 cm^−1^ [34].

On the other hand, the prepared rGO/Epoxy hardener composites at different percentages of rGO filler compound were confirmed by the identification of characteristic absorption peaks. The IR spectrum of the rGO/cured epoxy composites at 5 wt % displays strong absorption bands corresponding to O–H stretching vibrations at 3451.57 cm^−1^, C–H of CH_2_ stretching at 2924.42 cm^−1^, C=C stretching at 1605.89 cm^−1^, aromatic C–C stretching at 1490.93 cm^−1^, C–O–C stretching at 1228.66 cm^−1^, C–O stretching of ethers at 1040.64 cm^−1^, C–O–C stretching of the oxirane group at 820.33 cm^−1^, and rocking CH_2_ at 555.46 cm^−1^. A shifting of peaks is observed in the IR spectra when their blends have a strong interaction, such as hydrogen bonding or any another bonding.

### 3.4. Electromagnetic Properties

The electromagnetic properties of a material, namely the magnetic permeability (μ) and the electrical permittivity (ε), define the material’s response to electromagnetic waves. Permeability (μ = μ′ − jμ″) and permittivity (ε = ε′ − jε″) are complex numbers and a determinant on the nature of the material’s interaction with the magnetic and electrical fields of the wave, respectively. These interactions have two parts, namely: the storage and dissipation of energy parts. The storage of energy is due to the lossless energy exchange between the material and the real parts of the field. The dissipation of energy happens when material’s absorbed electromagnetic energy is converted to imaginary heat parts [35,36]. The loss tangent (tanθ) that is commonly used to describe dielectric losses is calculated by using tan$\theta =\frac{\varepsilon_{r}^{\prime \prime }}{\varepsilon_{r}^{\prime }}$.

#### 3.4.1. The Dielectric Properties of the rGO/Cured Epoxy Composites

**Figure 6:**
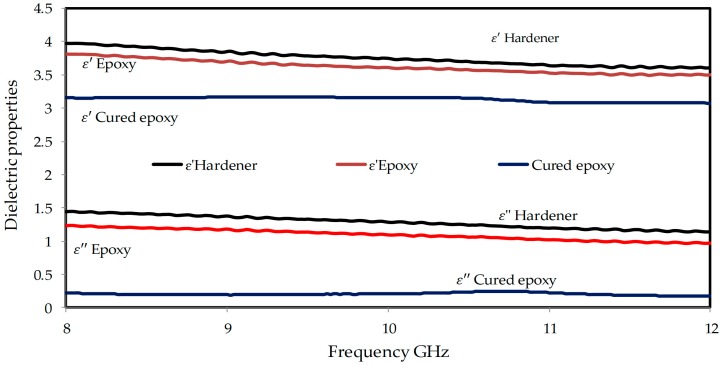
Relative permittivity for the epoxy, the hardener, and the cured epoxy at the X-band frequency.

In order to investigate the intrinsic reasons for the EMI shielding effectiveness of the composites, the dielectric properties of the epoxy resin, the hardener, the cured epoxy, and the rGO/cured epoxy composites at different percentages of rGO loading at the frequency range of 8–12 GHz (X-band frequency) were measured. Figure 6 illustrates the results of the dielectric properties for the epoxy, the hardener, and the cured epoxy (90% epoxy: 10% hardener) in the frequency range of 8–12 GHz. It can be observed that the ε′ and ε″ decreased for all samples as the frequency increased. Figure 6a,b shows that the hardener has the highest values of ε′ and ε″ of 3.94 and 1.48 at 8 GHz, then it progressively decreased to 3.63 and 1.39 at 12 GHz, respectively. Then, the ε′ and ε″ values of the epoxy decreased from 3.67 and 1.36 at 8 GHz to 3.42 and 0.98 at 12 GHz, respectively. The values of ε′ and ε″ decreased from 3.23 and 0.21 to 2.99 and 0.15, respectively, i.e., the dielectric properties of the cured epoxy decreased by increasing the frequency. This decrease can be attributed to the interfacial dipoles possessing less time to align themselves in the direction of the external field. In addition, the molecules are able to have a complete orientation at low frequencies but they are unable to have the same orientation at medium frequencies.

At very high frequencies, the molecules do not have enough time to orient themselves in the direction of the alternating field [37]. Furthermore, the reduction of the value of complex permittivity can be explained by the conductive and capacitive properties of the liquid form of the epoxy resin, which are induced by the ions and dipoles. When the liquid form of the epoxy resin is exposed to an external electric field, ions and dipoles easily align themselves because of the mobility acquired by the low viscosity of the liquid form. Also, the curing causes a decline in the number of dipolar groups, which decreases the loss factor and the dielectric constant. The relation between the viscosity and the dielectric constant is inversely proportional [38]. It was found that both the real and imaginary part of the dielectric constant, which regularly decreased as the curing reaction proceeded, were mainly affected by the disappearance of specific dipolar species, whose relaxation time did not change significantly.

**Figure 7:**
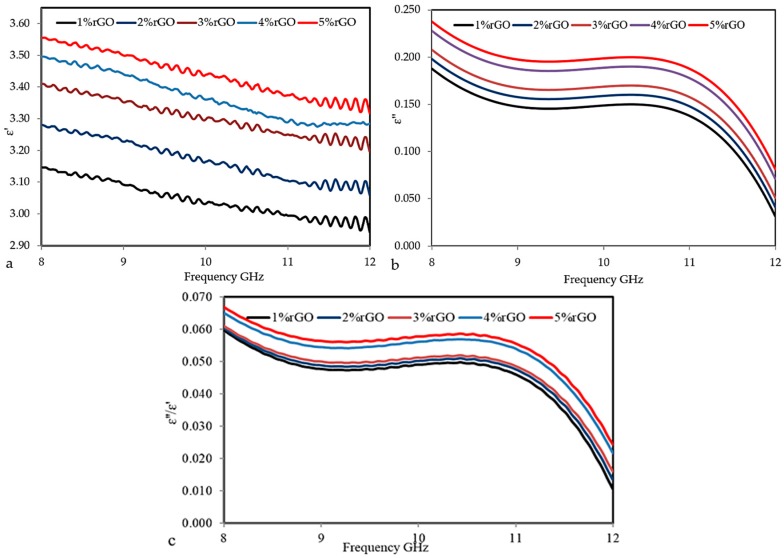
The frequency dependence of (**a**) The real part of permittivity (ε′), (**b**) The imaginary part of permittivity (ε″), and (**c**) The tangent loss (tanδ) at various rGO loadings.

The effects of filler functionality and volume loading on the dielectric properties of the rGO/cured epoxy composites were studied. Figure 7a–c shows the dependence of the ε′, ε″, and tanδ for the rGO/cured epoxy composites at different percentages of filler on the frequency. These figures show that, as more rGO particles were added to the epoxy matrix, the dielectric constant and loss factor gradually increase, with a high dielectric constant but a low loss factor comparable to that of a neat epoxy. The dielectric permittivity increment can be described as interfacial polarization, also known as the Maxwell–Wagner–Sillars (MWS) effect. In the composites, at 1% rGO, there was little distribution of rGO 88–92 particles; thus, there is a weak interaction with the matrix. When the rGO concentration is raised, clusters of filler particles are formed. A cluster may be considered as a region in the epoxy matrix where the particles are in contact or very close to each other as illustrated in the results of the scanning electron microscopy (SEM).

The average polarization that is associated with a cluster is larger than that of an individual particle because of an increase in the dimensions of the composite inclusion and, hence, greater interfacial area [39], which leads to a greater average polarization and, thus, a greater contribution to the dielectric permittivity. On the other hand, Figure 7a–c shows the dependence of the dielectric constant, loss factor, and loss tangent for the rGO/epoxy composites on the frequency. The dielectric constant of the composites decreases with the increase in the frequency. Due to the relatively high ε′ of rGO, the ε′ of the composites increased slightly from 3.15 for 1 vol % to 3.5 for 5 vol %. Furthermore, due to the relatively high ε″ of rGO, the ε″ of the composites increased gradually from 0.198 for 1 vol % to 0.246 for 5 vol %. In Figure 7c, it can be seen that the values of the loss tangent (tanδ) increase sequentially with increasing amounts of rGO in the composites, and were found to be 0.059 for 1 wt %, and 0.067 for 5 wt %. It could be obviously observed that the *E*′, *E*″, and tanδ of the rGO composites with different volume fractions decreased as the frequency increased from 8 to 12 GHz. The result is due to the space-charge polarization, which originates from the conductor–insulator interfaces.

#### 3.4.2. EMI Shielding Effectiveness

**Figure 8:**
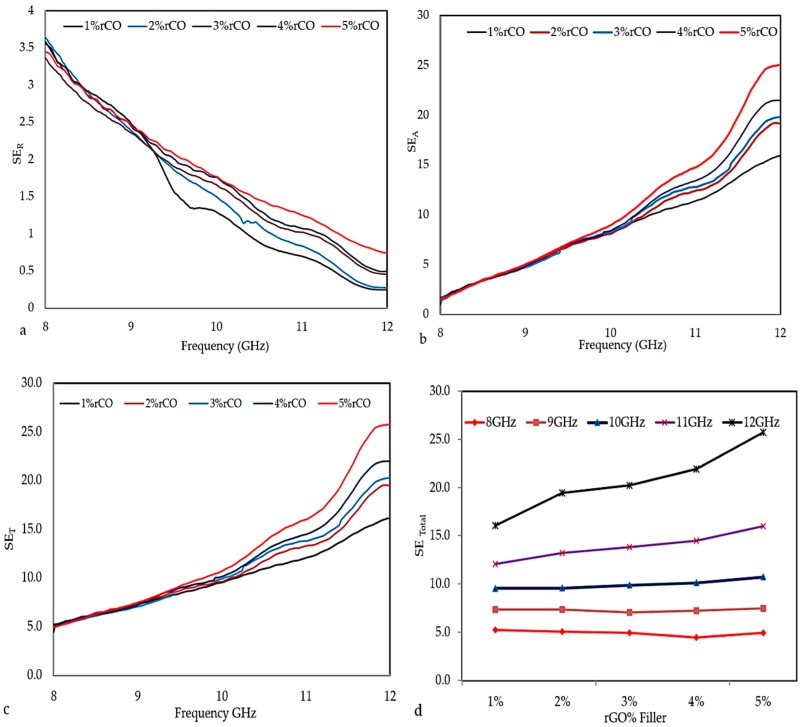
The Shielding Effectiveness (SE) (**a**) SE_R_, (**b**) SE_A_, and (**c**) SE _total_ for 6-mm-thick rGO/cured epoxy composites over the X-band. (**d**) The electromagnetic interference (EMI) SE _Total_ of composites with various percentages of filler at selected frequencies.

Figure 8a–d shows the SE_R_, SE_A_, and SE _total_ values of the composites with different percentage fractions of rGO loading. Figure 8a shows that the SE_R_ values of the rGO/cured epoxy composites decreased in frequency from 3.59 dB at 8 GHz to 0.45 dB at 12 GHz. The SE_A_ values of the rGO/cured epoxy composites increase significantly as shown in Figure 8b. Clearly, the SE_A_ values of the composites increased from 1.32 dB at 8 GHz to 24.78 dB at the 12 GHz frequency. Moreover, the SE_A_ value was enhanced with an increase in frequency, while the SE_R_ values decreased obviously. Based on the results, it can be concluded that absorption was the primary EMI shielding mechanism. The electron motion hysteresis in these dipoles under an alternating electromagnetic field induced additional polarization relaxation process which were favourable in enhancing microwave absorption attenuation [40]. Therefore, the rGO filler mixture with cured epoxy made a contribution to improving the EMI SE absorbing ability of the composites.

The SE _total_ values of the rGO/cured epoxy composites are shown in Figure 8c. The results confirm that the addition of rGO to the matrix improved the EMI shielding property of the composites, which increased with increasing the rGO content. This result can be attributed to the increase in the polarity of all blends due to the increase of rGO concentration, which led to the improvement of the shielding ability by vast numbers of mobile charge carriers (electrons or holes) that made the major mobility interaction with an external EM field possible and easier. Therefore, adding rGO to the matrix lead to the convergence of the composite particles with each other, which facilitates the process for moving mobile charge carriers that could move freely along this convergence [41]. The SE _total_ values of the composites with 1%, 2%, 3%, 4%, and 5% mass fractions of rGO at 8, 9, 10, 11 and 12 GHz are presented in Table 2. 

**Figure 9:**
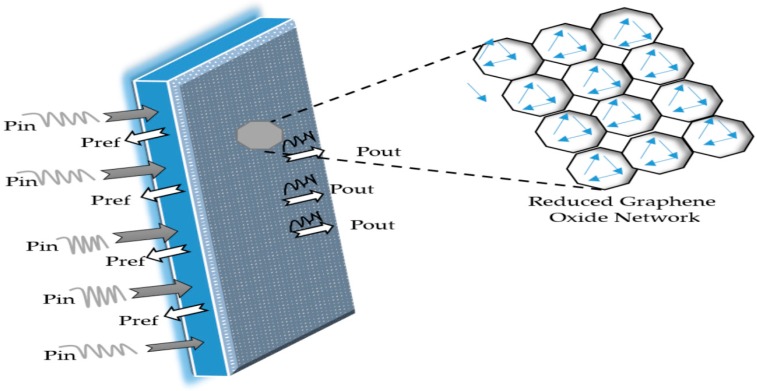
A schematic representation of microwave transfer across the rGO/cured epoxy composite with a thickness of 6 mm.

We now discuss the surface chemistry of rGO and the reinforcing mechanisms of rGO in the polymer matrices. The rGO nanoparticles were distributed uniformly throughout the whole cured epoxy matrix. This distribution can greatly enhance the utilization ratio of rGO, leading to a reduced rGO loading and an increase in the electrical conductivity of the composite. More importantly, densely packed graphene networks at the interfaces can effectively interact with incident radiation, leading to a very high EMI SE. To better understand the shielding mechanism, the rGO/cured epoxy composite can be considered as a “skin” that is composed of closely packed cells, with dense rGO layers as highly conductive “membranes”. As shown schematically in Figure 9, incident electromagnetic microwaves entering the “skin” are attenuated by reflecting, scattering, and adsorption many times by the multiple layers of membranes. The “cells” of the rGO/cured epoxy composite lead to a great number of membranes such that it is very difficult for waves to penetrate this functional skin.

#### 3.4.3. Cole–Cole Plot Analysis

The dielectric relaxation as a whole is the result of the movement of dipoles (dielectric relaxation) and electric charges (ionic relaxation) due to an applied alternating electric field. The Debye relaxation model has been widely employed to describe the response of molecules to an applied field. The rGO with a lower loss factor (ε″) has a stronger microwave absorption for three reasons: First, based on previous reports [42], apart from magnetic loss and dielectric loss, another concept of importance relating to the absorption of microwaves is the impedance match characteristic. Having an absorber permittivity that is too high harms the impedance match, resulting in weak absorption and strong reflection [43]. That explains the reasons for the exhibition of stronger microwave absorption by low-permittivity rGO. The second reason is that electronic spin is involved in the microwave band energy transition, which means that for microwave absorption, there is a requirement for greater spin states.

Near the Fermi level as documented, localized states could be created via the introduction of lattice defects. Additionally, electromagnetic energy absorption by a transition to the Fermi level from contiguous states can take place on the absorber surface when irradiation is incident on it [44]. Thus, the existence of defects in rGO favours the absorption of electromagnetic energy, which is an additional reason for the rGO’s exhibition of a better microwave absorption ability. The third reason is the dielectric loss material’s electromagnetic wave absorption mechanism, which arises from the process of relaxation. According to the expression of the equation for Debye relaxation in its simplest form, a single relaxation time (τ) was assumed for the complex permittivity, which can be written using the equation as expressed in [45,46]

(6) $$\varepsilon_{r}=\varepsilon_{\infty }+\frac{\varepsilon_{s}-\varepsilon_{\infty }}{1+j2\pi f\tau }=\varepsilon^{\prime }-j\varepsilon^{\prime \prime }$$

where $\varepsilon_{s}$, $\varepsilon_{\infty }$, *f*, and τ are the static dielectric constant and the dielectric constant at infinite frequency, the frequency, and polarization relaxation time, respectively. Thus, $\varepsilon^{\prime }$ and $\varepsilon^{\prime \prime }$ can be described by

(7) $$\varepsilon^{\prime }=\varepsilon_{\infty }+\frac{\varepsilon_{s-}\varepsilon_{\infty }}{1+w^{2}\tau^{2}}$$

(8) $$\varepsilon^{\prime \prime }=\frac{w\tau \left(\varepsilon_{s-}\varepsilon_{\infty }\right)}{1+w^{2}\tau^{2}}.$$

Based on Equations (4) and (5), the relationship between $\varepsilon^{\prime }$ and $\varepsilon^{\prime \prime }$ can be deduced as

(9) $${\left(\varepsilon^{\prime }-\frac{\varepsilon_{s-\varepsilon_{\infty }}}{2}\right)}^{2}+{\left(\varepsilon^{\prime \prime }\right)}^{2}={\left(\frac{\varepsilon_{s-\varepsilon_{\infty }}}{2}\right)}^{2}.$$

**Figure 10:**
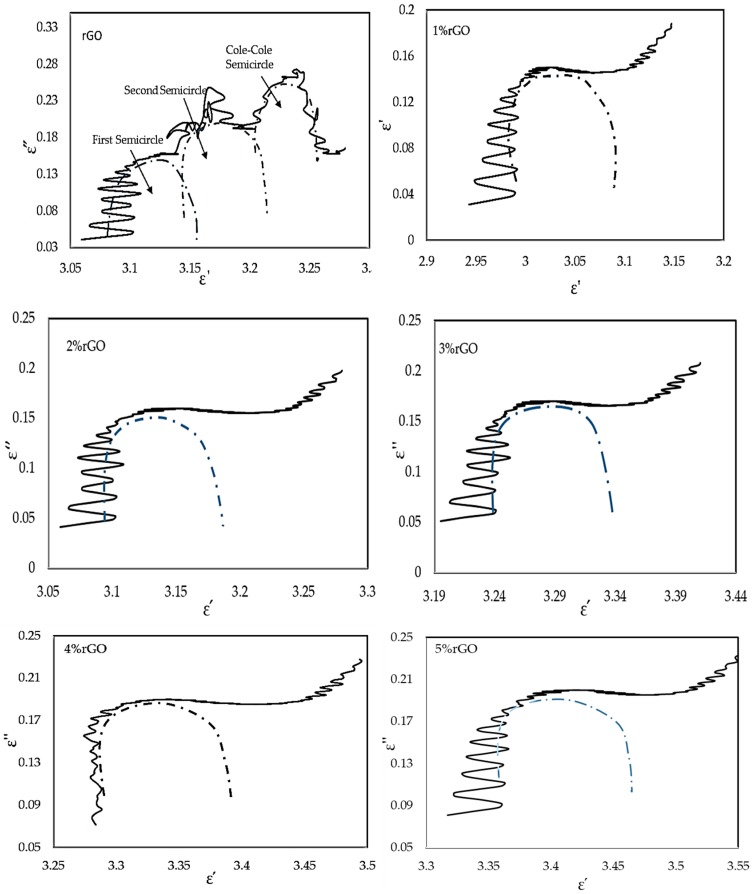
Typical Cole–Cole semicircles between the real part ($\varepsilon^{\prime }$) and the imaginary part ($\varepsilon^{\prime \prime }$) for the rGO and the cured rGO/epoxy in the frequency range of 8–12 GHz.

Figure 10 presents the ε′ versus ε″ curve characteristic, which is also called the Cole–Cole semicircle [47]. The figures with a clear segment present the rGO’s three overlapped Cole–Cole semicircles, but only a single semicircle at the different filler percentages for the rGO/cured epoxy. This suggests that for rGO/cured epoxy and tripartite relaxation processes, there is a sole relaxation process for rGO, with one Debye relaxation process assigned to each semicircle. For rGO/cured epoxy, the sole process of relaxation may appear as follows: under the lag of induced charges that counters the externally applied field that results in the relaxation, the alternating electromagnetic field converts the electromagnetic energy to heat energy, so the attenuation of microwaves occurs [48,49]. In the rGO/cured epoxy, due to many delocalized electrons, this process of dielectric relaxation is obvious, and a big Cole–Cole semicircle protrudes, as shown in Figure 10, for different percentages of filler. Hence, the major reason for the microwave absorption of the rGO/cured epoxy is the dielectric relaxation.

The presence of groups and residual defects in rGO is well-known [50]. As the rGO is reconstructed, so is the process of dielectric relaxation that is caused by the occurrence of motivating charges’ lateness, just as in the case of graphite. However, the case here is not as clear as it is in the case of graphite, due to the existence of a disrupted grapheme lattice. Thus, the size of its corresponding Cole–Cole semicircle becomes smaller. So, the two processes of relaxation of rGO clearly emerge from groups and defects, which can be explained as follows: first, the defects can act as polarization centres, with the ability to generate polarization relaxation, which would then attenuate electromagnetic wave and electromagnetic fields, resulting in a thorough microwave loss effect [51]. Second, the presence of chemical bonds that contain oxygen means that the electron catching ability differs between the oxygen atom and carbon atom, resulting in polarization of the electric dipole. Thus, more polarization relaxation is encouraged by the motion hysteresis of the electrons in these dipoles under the influence of an alternating electromagnetic field, which supports the enhancement of the microwave absorbing ability.

## 4. Conclusions

The preparation of rGO was carried out using an ammonia solution method. Cured epoxy composites homogenized with the produced rGO at different loadings have been successfully fabricated using a mini-mechanical vortex mixer. The effect of the rGO powder and their loading on the dielectric properties and mechanical properties was investigated and compared. Various characterizations, including FT-IR, XRD, and FE-SEM, were performed on the samples. The FE-SEM images showed the layered and porous structure of rGO. The dielectric properties of the rGO/cured epoxy composites were measured in the frequency range from 8 to 12 GHz. The rGO in the cured epoxy matrix, even at the lowest concentration of 1%, has been found to show low values of dielectric properties. The calculated shielding effectiveness value of the composite with 5% rGO by weight in the cured epoxy matrix is quite high, i.e., 25.748 dB at 12 GHz at a thickness of 6 mm. The Cole–Cole plots showed the presence of only one process of dielectric relaxation for the rGO/cured epoxy composites with a poor impedance match characteristic, which resulted from the weak ability for microwave absorption. However, the groups and residual defects in rGO can not only enhance the impedance match characteristic, but also introduce a transition from contiguous states to the Fermi level, polarization relaxation of electronic dipole groups, and defect polarization relaxation, all of which favours electromagnetic wave absorption and penetration.

## Figures and Tables

**Table 1:** The compositions of the nanocomposites.

Sample	wt % rGO	wt % Cured Er	Mass (gm) rGO	Mass (gm) Cured Epoxy	Mass (gm) Er	Mass (gm) H	Mass (gm) rGO/Cured Epoxy
rGO + cured epoxy	1	99	0.05	4.95	4.455	0.495	5 gm
	2	98	0.1	4.9	4.41	0.49	
	3	97	0.15	4.85	4.365	0.485	
	4	96	0.2	4.8	4.32	0.48	
	5	95	0.25	4.75	4.275	0.475	

Er, epoxy resin; H, hardener.

**Table 2:** The SE _total_ (dB) values for the rGO composites dependent on the frequencies and percentages of filler.

Freq (GHz)	1 wt %	2 wt %	3 wt %	4 wt %	5 wt %
8	5.233	5.061	4.912	4.451	4.927
9	7.309	7.322	7.038	7.239	7.476
10	9.516	9.556	9.872	10.109	10.692
11	12.038	13.226	13.779	14.444	15.991
12	16.046	19.434	20.226	21.933	25.748
